# Maternal Obesity Modulates Postpartum Inflammatory and Hormonal Profiles, Without Detectable Differences in Tested Redox Markers

**DOI:** 10.1111/aji.70227

**Published:** 2026-03-17

**Authors:** Juliana Augusta Dias, Isabela Carvalho Guimarães, Vinícius Lopes Cantuária, Bruna Oliveira Costa, Joyce Mirlane Moreira Costa, Bruna Caroline Chaves Garcia, Juliane Duarte Santos, Lourdes Fernanda Godinho, Marina Luiza Baêta Costa, Etel Rocha‐Vieria, Marco Fabrício Dias‐Peixoto, Kinulpe Honorato‐Sampaio

**Affiliations:** ^1^ Faculdade De Medicina da Universidade Federal Dos Vales do Jequitinhonha e Mucuri Diamantina Minas Gerais Brazil; ^2^ Programa De Pós‐Graduação em Ciências da Saúde da Universidade Federal Dos Vales do Jequitinhonha e Mucuri Diamantina Minas Gerais Brazil; ^3^ Departamento de Educação Física Faculdade De Ciências Biológicas e Da Saúde da Universidade Federal Dos Vales do Jequitinhonha e Mucuri Diamantina Minas Gerais Brazil

**Keywords:** delivery, inflammation, leucocytes, parturition, placenta, steroid hormones

## Abstract

**Problem:**

Maternal obesity is associated with elevated inflammatory markers, hormonal dysregulation, and metabolic disturbances. However, how maternal obesity modulates systemic inflammatory, hormonal, and redox profiles in the peripartum and postpartum periods remains incompletely understood.

**Method of Study:**

This observational study evaluated inflammatory biomarkers, steroid hormone levels, and oxidative stress markers in obese (*n* = 8) and non‐obese (*n* = 11) pregnant women undergoing labor induction. Peripheral maternal blood samples were collected immediately before induction and again within approximately 5–10 mins after delivery. Placental tissue was collected postpartum within the same interval. Comparative analyses between groups and time points were performed using repeated‐measures statistical models.

**Results:**

C‐reactive protein (CRP) levels increased postpartum only in the obese group, indicating an enhanced inflammatory response after delivery. In contrast, interleukin‐6 (IL‐6) levels declined postpartum only in the non‐obese group. No between‐group differences were detected in the oxidative stress markers assessed, either in maternal blood or placental tissue.

**Conclusions:**

Maternal obesity is associated with distinct postpartum inflammatory and hormonal profiles, characterized by sustained CRP elevation and altered cytokine dynamics. No between‐group differences were detected in the redox markers assessed, suggesting preserved redox balance in the parameters evaluated. These findings highlight the importance of considering postpartum immune modulation in obese parturients and support further investigation into obesity‐associated inflammatory regulation during the peripartum period.

## 1 Introduction

Obesity is a medical condition characterized by excessive accumulation of adipose tissue (AT) and is commonly assessed using body mass index (BMI), calculated as weight in kilograms divided by the square of height in meters. According to the World Health Organization (WHO), individuals with a BMI of ≥30 kg/m^2^ are classified as obese [1]. Its etiology is multifactorial, involving genetic, behavioral, environmental, and socioeconomic influences [2].

Obesity is associated with chronic low‐grade inflammation and elevated circulating levels of inflammatory markers, contributing to the development of several diseases [3]. AT functions not only as an energy reservoir but also as an active endocrine organ, secreting adipokines and cytokines. These mediators regulate lipid metabolism, insulin sensitivity, and immune responses [4]. AT exists primarily in two forms: white (WAT), which constitutes the bulk of AT and surrounds major organs and vessels, and brown (BAT), which is metabolically active but comprises only about 4.3% of total AT in adults [5]. Obesity induces hypertrophy and dysfunction of WAT adipocytes and promotes immune cell infiltration, leading to an inflammatory microenvironment that contributes to systemic metabolic dysregulation [6].

A growing number of women are overweight or obese at the onset of pregnancy, mirroring global trends. Maternal obesity is a major risk factor for adverse outcomes, being associated with oxidative stress, immune dysfunction, and chronic inflammation [7]. It also increases the likelihood of complications such as gestational diabetes and preeclampsia, often necessitating medical interventions including cesarean delivery and labor induction [8], contributing to metabolic abnormalities such as oxidative stress, immune dysfunction, and chronic inflammation. Furthermore, excessive maternal adiposity complicates clinical management, affecting procedures such as epidural anesthesia, fetal ultrasonography, and surgical delivery [9].

In addition to maternal risks, high maternal BMI is associated with unfavorable fetal outcomes, including preterm birth, macrosomia, congenital anomalies, and altered neurodevelopment. Long‐term consequences extend to offspring health, with increased risk of obesity, cardiovascular disease, type 2 diabetes, and cancer later in life [10, 11]. These intergenerational effects perpetuate a cycle of metabolic disease that may extend over three or more generations [12] imposing a growing economic burden on healthcare systems.

Obesity is marked by hyperinsulinemia, hyperlipidemia, hyperleptinemia, and chronic inflammation [13]. This inflammatory state, termed “metainflammation”, results from immune dysregulation and activation of pro‐inflammatory pathways [14]. Although pregnancy physiologically alters immune responses, maintaining a finely tuned balance between pro‐ and anti‐inflammatory cytokines is critical for successful implantation, placental development, and fetal tolerance. Maternal obesity disrupts this equilibrium, favoring a pro‐inflammatory intrauterine environment [15].

Circulating markers such as interleukin‐6 (IL‐6) and C‐reactive protein (CRP) are typically elevated in individuals with increased adiposity [16], whereas lean individuals exhibit higher levels of anti‐inflammatory cytokines like IL‐10 [17]. Pregnancy itself is associated with gestational‐age‐dependent shifts in the inflammatory milieu, including a rise in neutrophil count. Maternal obesity exacerbates these changes and is linked to increased reactive oxygen species (ROS) production and oxidative stress [18]. The interaction between metabolic dysfunction, inflammation, and oxidative signaling, especially in the placenta, plays a critical role in mediating adverse maternal and fetal outcomes [19].

The cellular antioxidant defense system maintains redox homeostasis through enzymatic antioxidants (e.g., superoxide dismutase [SOD], catalase [CAT], and glutathione peroxidase [GPx]) and non‐enzymatic molecules, which neutralize excess ROS [20]. Oxidative stress arises when this balance is disrupted in favor of oxidants, leading to cellular and molecular damage. Biomarkers of oxidative injury provide measurable evidence of this imbalance [21]. Inflammation and oxidative stress are thus intimately linked, as activated immune cells generate ROS during the inflammatory response [22].

Inflammatory and oxidative stress biomarkers play essential roles in pregnancy physiology and the labor process. While transient inflammation is necessary for successful parturition, maternal obesity may provoke a persistent inflammatory state that undermines this adaptive response. Although maternal obesity is strongly associated with adverse pregnancy and labor outcomes, less is known about how obesity modulates physiological inflammatory and hormonal responses during the peripartum period in the absence of overt obstetric pathology. In addition, there is a paucity of studies evaluating both systemic and placental biomarkers in this context. Therefore, this study aimed to evaluate inflammatory and oxidative stress biomarkers in maternal blood and placental tissue from pregnant women with and without obesity undergoing labor induction.

## 2 Methods

### 2.1 Participants

Between January and October 2023, a cross‐sectional quantitative study was conducted at Hospital Nossa Senhora da Saúde, located in Diamantina, Minas Gerais, Brazil. Nineteen pregnant women admitted for labor induction were invited to participate. Sample size estimation was based on a previous study [23], which reported neutrophil counts of 8,800 ± 2,200/mm^3^ in women without labor and 15,300 ± 5,000/mm^3^ immediately after delivery. Using OpenEpi version 3.01, with a power of 90% and an alpha of 0.05, the minimum required sample size was determined to be eight participants per group.

All participants provided written informed consent. The study protocol was approved by the Ethics Committee of the Federal University of Vales do Jequitinhonha and Mucuri (protocol number: 58690622.90000.5108).

Inclusion criteria were: (1) age ≥18 years; (2) ability to communicate verbally in Portuguese; (3) confirmed term pregnancy by first‐trimester ultrasound; (4) nulliparity; (5) intact membranes; and (6) cephalic presentation. Exclusion criteria included: multiple gestation; labor complications (e.g., fetal distress, meconium‐stained amniotic fluid, dystocia, or any indication for intrapartum cesarean section other than induction failure); cervical cerclage; medication use during data collection (other than labor inducers such as misoprostol or oxytocin); and the presence of pre‐existing or gestational comorbidities.

Labor induction was chosen as a methodological strategy to standardize the timing of biological sample collection and reduce variability related to spontaneous labor onset. All included women underwent induction for non‐pathological indications, according to institutional clinical practice. These indications included post‐term pregnancy, elective induction upon maternal request, social or logistical considerations (such as long geographic distance from the hospital or limited availability of a support person), and previous adverse obstetric history. Women with medical or obstetric complications were excluded to minimize confounding related to pathological inflammatory or metabolic conditions.

Participants were classified into two groups based on their BMI: Obese group: women with BMI ≥30 undergoing labor induction; Non‐obese group: women with BMI <30 undergoing labor induction.

Clinical data were obtained through a structured questionnaire, including maternal age, gestational age, BMI in the first and third trimesters, BMI delta, time from induction to pain onset, induction‐to‐delivery interval, duration of labor, misoprostol and oxytocin dosages, and mode of delivery (vaginal or cesarean).

Initial assessments were performed at hospital admission. Eligible participants received detailed study information and were invited to enroll. The first biological samples were collected prior to labor induction. All included women had a gestational age ≥37 weeks, were not in active labor (i.e., absence of uterine contractions), and underwent induction with misoprostol and/or oxytocin according to institutional protocols.

Total neutrophil count, CRP and IL‐6 were defined a priori as primary outcomes. All other hematological, hormonal, and oxidative stress markers were considered secondary or exploratory endpoints.

### 2.2 Sample Collection

Peripheral maternal blood was collected immediately before labor induction and again immediately after delivery. Postpartum blood and placental tissue samples were obtained within approximately 5–10 min following fetal expulsion. Blood was drawn into tubes containing heparin or EDTA, stored at 2–8°C, and transported to a private laboratory for analysis. Assays included complete blood count, hormonal quantification (estradiol, estriol, progesterone), CRP, ferritin, cytokines (IL‐6, IL‐8, IL‐10), and oxidative stress profiles in leukocytes.

Placental tissue samples (∼1 cm^2^) were collected from a standardized site located approximately 3–5 cm from the umbilical cord insertion, avoiding visibly altered, infarcted, or calcified regions. Each sample included both the maternal (basal plate) and fetal (chorionic plate) surfaces, without separation or pooling of distinct lobules. Tissues were excised using sterile instruments and rinsed thoroughly in phosphate‐buffered saline (PBS) to minimize residual maternal blood contamination. Samples were then placed in labeled Eppendorf tubes, snap‐frozen in liquid nitrogen, and stored at −80°C until oxidative stress analysis.

### 2.3 Cytokine Quantification

Plasma cytokine levels were determined using enzyme‐linked immunosorbent assay (ELISA) kits, performed in duplicate according to manufacturer instructions. Commercial kits used were IL‐6 and IL‐10 (DuoSet, R&D Systems, Minneapolis, MN, USA) and IL‐8 (ImmunoTools, R&V Intertrade Co., Ltd., Thailand).

### 2.4 Redox State

Blood samples were centrifuged and leukocytes were isolated for oxidative stress analysis. The following redox parameters were measured: 1) Protein carbonyl derivatives [24]; 2) Thiobarbituric acid reactive substances (TBARS) [25]; 3) Total antioxidant capacity (TAC) via ferric reducing antioxidant power (FRAP) assay [26]. In placental tissue, the redox markers evaluated included: 1) Protein carbonyl derivatives; 2) CAT activity [27]; 3) SOD activity [28]; 4) TAC by FRAP. All procedures followed the methodology described by Rodrigues et al. (2021) [29]. All analyses were performed in triplicates and normalized to total protein content, determined by the Bradford method [30]. The intra‐assay coefficients of variation were below 10%.

### 2.5 Placental ROS

ROS in cryopreserved placental tissue was assessed using the fluorescent probe 2’,7’‐dichlorodihydrofluorescein diacetate (DCFH‐DA, D6883 Sigma), as described by Lopes Cantuária et al. (2025) [31]. Briefly, 10‐µm placental sections were mounted on glass slides and treated with 20 µL of 50 µM DCFH‐DA solution. Samples were incubated at 37°C for 20 min. Fluorescence intensity from dichlorofluorescein (DCF) was detected under a fluorescence microscope (Olympus BX53F) using a 10× objective and excitation wavelength of 492–495 nm. Measurements were taken at 0, 2, 4, and 6 min, and results were expressed as the area under the fluorescence intensity–time curve. This time window was selected because fluorescence signal became undetectable in several samples beyond this interval. Assay performance was previously validated in rat cardiac tissue, as reported in our prior publication [26], and negative controls were obtained by omitting DCFH‐DA incubation.

### 2.6 Statistical Analysis

Data are presented as mean ± standard error (SE). Normality was assessed using the Shapiro–Wilk test, and homogeneity of variances was evaluated using Levene's test. Categorical variables are presented as absolute and relative frequencies and were analyzed using Fisher's exact test. Comparisons between obesity groups (non‐obese vs. obese) were performed using Student's *t*‐test for normally distributed variables and the Mann–Whitney U test for non‐parametric data.

Longitudinal changes (pre‐induction vs. post‐delivery) and between‐group differences were analyzed using two‐way repeated‐measures ANOVA, with subject included as a matching factor. Main effects of time, group, and the time × group interaction were reported. Post hoc pairwise comparisons were conducted using the two‐stage linear step‐up procedure of Benjamini, Krieger, and Yekutieli to control the false discovery rate, applied specifically to within‐group comparisons across time points. Post hoc analyses were performed using GraphPad Prism (version 8.0.1). As this software does not provide confidence intervals for BKY‐adjusted comparisons, confidence intervals are not reported for these analyses.

Sensitivity analyses were performed using repeated‐measures ANOVA models adjusted separately for mode of delivery (vaginal vs. cesarean section) implemented in JASP (version 0.95.4). Others covariates were not included due to minimize the risk of model overfitting.

## 3 Results

A total of 19 pregnant women were included in the study, with 11 allocated to the non‐obese group and 8 to the obese group. Baseline characteristics showed no significant differences between groups in maternal age, gestational age, BMI variation during pregnancy, induction‐to‐pain interval, induction‐to‐delivery interval, labor duration, misoprostol dosage, maximum oxytocin dose, or mode of delivery (Table 1). However, first‐ and third‐trimester BMI values were significantly higher in the obese group (*p* < 0.001). No adverse fetal or neonatal outcomes were observed in either group. All newborns were delivered at term and presented with appropriate clinical conditions at birth.

**TABLE 1:** Comparison of characteristics of non‐obese and obese parturients.

Parameter	Non‐obese (*n* = 11)	Obese (*n* = 8)	*p*‐value	Test
Maternal age	25.09 ± 2.13	24.50 ± 1.51	0.837	T
Gestational age	39.37 ± 0.43	38.83 ± 0.45	0.399	T
BMI first trimester	23.56 ± 1.18	35.80 ± 2.15	< 0.001	T
BMI third trimester	28.01 ± 1.14	39.00 ± 2.29	< 0.001	T
BMI delta	4.45 ± 0.75	3.20 ± 0.53	0.223	T
Induction time to onset of pain	8.55 ± 2.98	12.33 ± 4.14	0.543	U
Time from induction initiation to delivery	12.00 ± 3.27	16.00 ± 3.26	0.264	U
Labor duration	5.09 ± 0.65	5.00 ± 1.27	0.944	T
Misoprostol dose	40.91 ± 13.60	59.38 ± 17.64	0.411	T
Maximum oxytocin dose	54.55 ± 20.68	73.50 ± 29.78	0.933	U
Outcome				
(Vaginal delivery vs. Cesarean section)	(7 vs. 4) (63,6% vs. 36,4%)	(6 vs. 2) (75% vs. 25%)	> 0.999	F

Values expressed as mean ± standard error and Bold values are significant *p* < 0.05.

F: Fisher's exact test; T: Student's t‐test; U: the Mann‐Whitney U test.

Hematological analyses revealed no significant differences in red blood cell parameters between groups. In contrast, the non‐obese group exhibited a significant increase in total leukocyte count during labor (*p* = 0.0039), driven primarily by increases in neutrophil count (*p* = 0.0023) and segmented cell count (*p* = 0.0318) following induction. These hematologic changes were not observed in the obese group (Table 2).

**TABLE 2:** Comparison of blood cell profile of non‐obese (*n* = 11) and obese (*n* = 8) parturients before labor induction (pre‐induction) and immediately after delivery (post‐delivery).

					*p*‐value			
Parameter	Groups	Pre‐induction	Post‐delivery	Δ Post–Pre (SE); *q*‐value	Pos hoc (BKY)	Effect of time	Effect of obesity	Interaction
Red blood cells (millions/mm3)	Non‐obese	4.41 ± 0,09	4.11 ± 0,19	−0.2955 (0.1440); 0.1174	0.0524	0.3675	0.8293	0.1005
	Obese	4.26 ± 0,14	4.35 ± 0,17	+0.0900 (0.0168); 0.6310	0.6009			
Hemoglobin (g/dL)	Non‐obese	13.11 ± 0,28	12.25 ± 0,67	−0.8636 (0.4459); 0.1461	0.0696	0.3657	0.4111	0.1316
	Obese	12.06 ± 0,33	12.29 ± 0,42	+0.2250 (0.5229); 0.7060	0.6724			
Hematocrit (%)	Non‐obese	40.20 ± 0.85	37.82 ± 2.01	−2.382 (1.303); 0.1795	0.0855	0.4565	0.3289	0.1263
	Obese	36.76 ± 1.12	37.61 ± 1.23	+0.8500 (1.529); 0.6149	0.5856			
Mean corpuscular hemoglobin (pg)	Non‐obese	29.69 ± 0,30	29.76 ± 0,44	+0.0727 (0.2102); 0.8430	0.7336	0.9752	0.0841	0.6815
	Obese	28.44 ± 0.68	28.38 ± 0.71	−0.0625(0.2465); 0.8430	0.8028			
Mean corpuscular volume (u^3^)	Non‐obese	91.06 ± 0.88	91.96 ± 1.27	+0.9000 (0.5301); 0.2263	0.1078	0.1561	**0.0208**	0.4818
	Obese	86.53 ± 1.66	86.84 ± 1.77	+0.3125 (0.6216); 0.6527	0.6216			
Mean corpuscular hemoglobin concentration (%)	Non‐obese	32.60 ± 0.09	32.36 ± 0.14	−02364 (0.1981); 0.4847	0.2493	0.1956	0.3566	0.8431
	Obese	32.84 ± 0.41	32.66 ± 0.27	−0.1750 (0.2323); 0.4847	0.4616			
Red blood cell distribution width (%)	Non‐obese	13.11 ± 0.29	13.05 ± 0.25	−0.0545 (0.1198); 0.6873	0.6546	0.9131	0.0875	0.4923
	Obese	14.29 ± 0,77	14.36 ± 0.68	+0.0750 (0.1404); 0.6873	0.6002			
Leukocytes (white blood cells)	Non‐obese	13400 ± 1691	20000 ± 2204	+6600 (1978); 0.0041	**0.0039**	**0.0018**	0.7788	0.5308
	Obese	15250 ± 2938	19900 ± 2982	+4650 (2319); 0.0321	0.0611			
Neutrophils	Non‐obese	9693 ± 1443	16954 ± 2117	+7261 (2032); 0.0025	0.0025	0.0011	0.8866	0.4742
	Obese	11248 ± 2746	16217 ± 2982	+4969 (2383); 0.0275	0.0524			
Bands (cells/mm^3^)	Non‐obese	136 ± 63	302 ± 71	+165.4 (87.47); 0.1590	0.0757	0.0736	0.7419	0.5909
	Obese	146 ± 105	237 ± 54	+91.58 (102.6); 0.4036	0.3844			
Segs (cells/mm^3^)	Non‐obese	9484 ± 1398	14125 ± 1982	+4641 (1984); 0.0669	**0.0318**	**0.0445**	0.9222	0.3990
	Obese	11071 ± 2634	13066 ± 2830	+1995 (2327); 0.4234	0.4032			
Eosinophilic (cells/mm^3^)	Non‐obese	229 ± 41	264 ± 67	+34.91 (65.23); 0.9544	0.5995	0.7988	0.6745	0.6686
	Obese	274 ± 28	266 ± 49	−8.875 (76.49); 0.9544	0.9090			
Lymphocytes (cells/mm^3^)	Non‐obese	2098 ± 206	2279 ± 346	+180.6 (274.1); 0.5446	0.5187	0.3009	0.6036	0.8348
	Obese	2242 ± 279	2512 ± 296	+270.1 (321.4); 0.5446	0.4124			
Monocytes (cells/mm^3^)	Non‐obese	510 ± 62	503 ± 41	−7.364 (73.58); 0.9675	0.9215	0.8467	0.5466	0.7483
	Obese	536 ± 98	565 ± 50	+29.62 (86.28); 0.9675	0.7355			
Platelets (/mm^3^)	Non‐obese	238091 ± 15524	229091 ± 14518	−9000 (13477); 0.9783	0.5132	0.6238	0.7493	0.7181
	Obese	243500 ± 26804	242125 ± 31046	−1375 (15803); 0.9783	0.9317			

Values expressed as mean ± standard error and Bold values are significant *p* < 0.05. Two‐way repeated‐measures ANOVA, followed by the Benjamini, Krieger, and Yekutieli (BKY) two‐stage linear step‐up procedure.

Hormonal analyses demonstrated a significant reduction in serum levels of estriol, estradiol, and progesterone after labor induction in both groups. Notably, prior to induction, progesterone levels were significantly higher in the non‐obese group compared to the obese group (*p* = 0.0106), suggesting that nutritional status may influence hormone production during pregnancy (Table 3).

**TABLE 3:** Comparison of hormonal profile, and oxidative stress and inflammatory biomarkers of non‐obese (*n* = 11) and obese (*n* = 8) parturients before labor induction (pre‐induction) and immediately after delivery (post‐delivery).

					*p*‐value			
Parameter	Groups	Pre‐induction	Post‐delivery	Δ Post–Pre (SE); *q*‐value	Pos hoc (BKY)	Effect of time	Effect of obesity	Interaction
Estriol (ng/mL)	Non‐obese	11.30 ± 0.47	6.04 ± 1.22	−5.262 (1.156); 0.0006	**0.0003**	**0.0001**	0.9871	0.3343
	Obese	10.44 ± 0.98	6.94 ± 1.55	−3.492 (1.355); 0.0206	**0.0196**			
Estradiol (pg/mL)	Non‐obese	14474 ± 525	10902 ± 1487	−10902 (3572); 0.0415	**0.0395**	**0.0036**	0.3786	0.6397
	Obese	13491 ± 1509	8743 ± 2370	−8743 (4749); 0.0415	**0.0217**			
Progesterone (ng/mL)	Non‐obese	298.3 ± 36.87	155.8 ± 24.80	−142.5(2075); < 0.0001	**< 0.0001**	**< 0.0001**	0.0663	**0.0321**
	Obese	185.1 ± 24.39	117.2 ± 21.22	−67.87 (24.33); 0.0132	**0.0126**			
Leukocyte TBARs^1^	Non‐obese	3.04 ± 0.68	3.49 ± 0.69	+1.299 (2.266); 0.9036	0.5745	0.6135	0.1698	0.8064
	Obese	4.79 ± 1.08	6.09 ± 2.54	+0.4520 (2.533);	0.8606			
Leukocyte protein carbonyl content (nmol/protein mg)	Non‐obese	129.2 ± 39.29	127.3 ± 38.24	−1.912 (63.70); >0,9999	0.9764	0.9002	0.6954	0.9314
	Obese	150.8 ± 57.30	140.6 ± 50.43	−10.26 (71.21); >0,9999	0.8871			
Leukocyte total non‐enzymatic antioxidant activity (FRAP)^2^	Non‐obese	162.3 ± 41.80	204.0 ± 35.97	+41.76 (39.41); 0.6405	0.3050	0.5482	0.5757	0.4357
	Obese	160.0 ± 19.53	154.5 ± 46.67	−5.498 (44.06); 0.9474	0.9022			
CRP (mg/L)	Non‐obese	8.436 ± 2.956	11.59 ± 3.410	+3.155(2.136); 0.0830	0.1581	**0.0036**	0.2157	0.1635
	Obese	12.08 ± 2.192	20.03 ± 4.977	+7.950 (2.505); 0.0058	**0.0056**			
Ferritin (ng/mL)	Non‐obese	20.77 ± 4.088	22.95 ± 5.273	+2.173 (1.462); 0.1658	0.1579	**0.0430**	0.8780	0.6629
0.5308	Obese	17.24 ± 5.138	21.06 ± 5.267	+3.267 (1.979); 0.1658	0.1196			
Interleukin 6 (pg/mL)	Non‐obese	3.797 ± 1.16	1.706 ± 0.16	−2.091 (0.9860); 0.1048	0.0499	0.1005	0.2817	0.2941
	Obese	4.51 ± 1.10	4.03 ± 1.65	−0.4868 (1.102); 0.6979	0.6647			
Interleukin 8 (pg/mL)	Non‐obese	36.12 ± 7.935	42.91 ± 6.807	+6.793 (7.182); 0.7524	0.3583	0.4440	0.9401	0.6404
	Obese	37.72 ± 12.94	39.38 ± 11.90	+1.663 (8.029); 0.8805	0.8386			
Interleukin 10 (pg/mL)	Non‐obese	7.69 ± 1.73	6.20 ± 1.07	−1.489 (1.890); 0.7559	0.4422	0.8033	0.6245	0.4369
	Obese	5.97 ± 1.09	6.74 ± 0.913	+0.7714 (2.113); 0.7559	0.7199			

Values expressed as mean ± standard error and Bold values are significant *p* < 0.05. Two‐way repeated‐measures ANOVA, followed by the Benjamini, Krieger, and Yekutieli (BKY) two‐stage linear step‐up procedure.

Regarding inflammatory mediators, CRP levels increased significantly in the obese group following delivery (*p* = 0.0056), suggesting an amplified inflammatory response in this group. In contrast, ferritin levels remained unchanged between groups and over time. Cytokine analysis showed a significant post‐delivery decrease in IL‐6 levels in the non‐obese group (*p* = 0.0499). However, no significant changes were observed in IL‐8 or IL‐10 levels in either group.

Sensitivity analyses adjusting for mode of delivery were performed for the primary inflammatory outcomes (neutrophils, CRP, and IL‐6). Adjustment for delivery mode did not materially change the direction or magnitude of the main effects. The effect of time remained significant for neutrophils and CRP, and became significant for IL‐6 after adjustment, indicating that obstetric outcome contributes to variability in cytokine dynamics during labor (Table S1).

**FIGURE 1:**
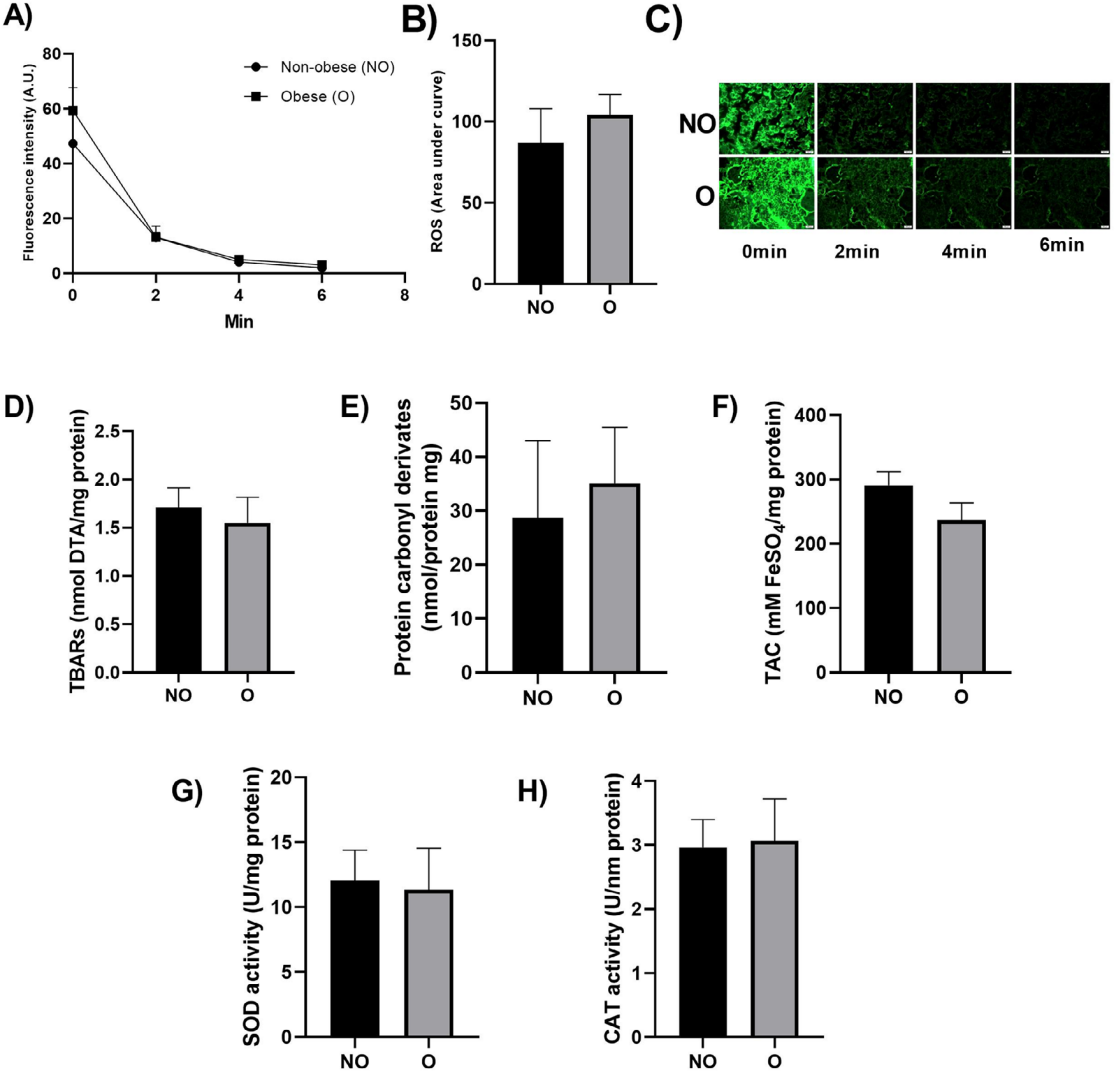
Placental redox status in parturients undergoing labor induction with and without obesity. (A) ROS production assessed by fluorescence microscopy using DCFH‐DA at 0, 2, 4, and 6 min. (B) Area under the fluorescence decay curve. (C) Representative images of placental samples during ROS production analysis. (D) TBARs levels. (E) Protein carbonyl derivatives. (F) TAC: Total non‐enzymatic antioxidant capacity. (G) SOD activity. (H) CAT activity. NO: Non‐obese; O: Obese. Data were analyzed using Student's *t*‐test, and no significant differences were observed.

Assessment of oxidative stress biomarkers in maternal blood, including TBARS, FRAP, and protein carbonyl content, showed no significant differences between groups or across time points. A similar pattern was observed in placental oxidative stress markers, including ROS production, TBARS, carbonyl protein levels, TAC, SOD, and CAT activity (Figure 1).

## 4 Discussion

This study demonstrates that maternal obesity is associated with distinct peripartum inflammatory and hormonal profiles when assessed longitudinally before labor induction and immediately postpartum. Using paired sampling, we observed that non‐obese women exhibited a significant leukocyte increase across the peripartum period, driven predominantly by neutrophil expansion, whereas this response was attenuated in obese parturients. In parallel, all participants showed a marked decline in circulating steroid hormones after delivery, while a postpartum increase in CRP was observed exclusively in the obese group. IL‐6 levels declined postpartum only among non‐obese women, indicating differential inflammatory dynamics associated with maternal adiposity.

To our knowledge, this is the first study to examine inflammatory and hormonal biomarkers in obese and non‐obese pregnant women at two tightly controlled and clinically relevant time points, immediately before labor induction and shortly after delivery. This design allowed us to characterize temporal changes in immune‐endocrine markers while minimizing confounding from interindividual variability and labor‐associated physiological fluctuations. By applying a standardized sampling protocol and enrolling women without overt pregnancy complications, we aimed to isolate the contribution of maternal obesity to peripartum inflammatory profiles rather than to obstetric pathology.

Importantly, the novelty of our findings extends beyond the paired study design. Our data indicate that maternal obesity is associated with altered inflammatory trajectories across the peripartum period, particularly in the immediate postpartum phase. Rather than reflecting a uniformly heightened inflammatory state, obesity appears to modulate the resolution of inflammation following delivery. This is supported by the selective postpartum increase in CRP among obese women and the absence of a concomitant decline in IL‐6, contrasting with the pattern observed in non‐obese parturients. Given the central role of IL‐6 in linking metabolic inflammation and labor‐associated immune activation, the persistence of IL‐6 levels postpartum in obese women, together with elevated CRP, suggests a sustained low‐grade inflammatory milieu characteristic of obesity. Notably, these immune alterations were observed in the absence of adverse obstetric or neonatal outcomes, underscoring that obesity‐related inflammatory modulation may occur independently of overt clinical complications and should be interpreted within the scope of the biomarkers assessed.

The leukocyte increase observed in non‐obese women is consistent with the well‐established rise in total leukocyte counts during pregnancy and labor, driven predominantly by neutrophils, while other white blood cell subtypes remain relatively stable in late gestation [32]. In contrast, obese women did not exhibit a significant postpartum leukocyte increase. This finding aligns with the concept that obesity, characterized by chronic low‐grade inflammation, may be associated with a persistently elevated inflammatory baseline [33]. Such a background inflammatory state may attenuate the magnitude of labor‐associated leukocyte changes, potentially reflecting altered inflammatory dynamics rather than an absence of immune activation.

CRP, a classical acute‐phase reactant, increased significantly postpartum only in obese women, reinforcing the association between obesity and heightened systemic inflammation during the peripartum period [34, 35, 36]. While CRP is widely used as a marker of inflammation, it is also linked to cardiometabolic risk [37], raising considerations regarding postpartum inflammatory burden in this population. Importantly, the selective increase in CRP among obese parturients supports the notion of altered inflammatory resolution following delivery, rather than a uniformly exaggerated inflammatory response.

IL‐6, a central mediator at the interface of metabolic inflammation and labor‐associated immune activation, declined significantly postpartum only in the non‐obese group. Obesity is characterized by elevated circulating cytokines, particularly IL‐6, which plays a key role in immune regulation and acute‐phase signaling [33]. Although normal pregnancy is associated with progressive cytokine elevation [38], and obesity may amplify this trend [39]. Although IL‐6 physiologically increases during labor [40], the postpartum decline of IL‐6 observed in non‐obese women may reflect a more efficient resolution of inflammatory signaling following delivery. In contrast, persistently elevated IL‐6 levels in obese women are consistent with a sustained low‐grade inflammatory milieu driven by excess adiposity [41, 42]. Although the unadjusted analysis did not demonstrate a significant main effect of time on IL‐6 levels, differences were identified in post hoc comparisons. Notably, sensitivity analyses adjusting for mode of delivery revealed a significant temporal effect on IL‐6, indicating that obstetric outcome contributes to variability in cytokine responses and may partially mask peripartum inflammatory dynamics in unadjusted models. Given that cesarean delivery is associated with distinct inflammatory and stress‐related responses compared with vaginal birth, this adjustment likely refines the detection of IL‐6 modulation rather than introducing spurious associations. These findings support the role of IL‐6 as a sensitive marker of peripartum inflammatory trajectories within the scope of the biomarkers assessed.

The postpartum decline in progesterone and estrogen levels observed in both groups is consistent with the endocrine shift associated with placental separation and the transition from pregnancy to the postpartum state. Progesterone plays a critical role in maintaining uterine quiescence, and its withdrawal at term permits the upregulation of pro‐inflammatory mediators and uterine activation pathways [43, 44, 45]. Notably, progesterone concentrations prior to labor induction were significantly lower in obese women, suggesting that maternal obesity may influence endocrine regulation during late pregnancy and potentially modify immune‐endocrine interactions during the peripartum period. Previous studies have shown that inflammatory and endocrine pathways are tightly interconnected during pregnancy and the peripartum period. Steroid hormones such as progesterone and estrogens contribute to immune tolerance and uterine quiescence, whereas their functional withdrawal near term coincides with activation of inflammatory signaling pathways required for parturition [46, 47, 48]. Conversely, inflammatory cytokines can influence steroid hormone synthesis and signaling, highlighting the bidirectional crosstalk between immune and endocrine systems. The concurrent assessment of hormonal and inflammatory markers in this study therefore provides an integrated view of peripartum immune‐endocrine regulation and suggests the crosstalk between immune and endocrine systems.

Although obesity is strongly associated with increased oxidative stress, impaired antioxidant defenses, and mitochondrial dysfunction [49, 50, 51], no between‐group differences were detected in the ROS or oxidative stress markers assessed in maternal blood or placental tissue. These findings indicate that, within the scope of the markers evaluated, maternal obesity was not associated with detectable alterations in redox status during the peripartum period. One plausible interpretation is that adaptive physiological mechanisms during pregnancy may contribute to the maintenance of redox balance despite underlying metabolic disturbances. In this context, the placenta may play a central role in buffering oxidative fluctuations and preserving a relatively stable oxidative environment at the maternal–fetal interface, even in pregnancies complicated by obesity [52, 53]. Together, these observations underscore the complexity of redox regulation during pregnancy and highlight the need for further studies employing broader and dynamic redox assessments to better characterize maternal‐fetal adaptations across different metabolic conditions.

Some limitations of this study should be acknowledged. First, inflammatory biomarkers were assessed in maternal circulation, whereas inflammatory markers were not evaluated in placental tissue, precluding a more detailed characterization of immune signaling at the maternal–fetal interface. Given the immunological complexity of the placenta, future studies incorporating placental and fetal membrane inflammatory analyses may provide additional insight into compartment‐specific immune regulation in the context of maternal obesity. Second, the inflammatory profile was characterized using a focused cytokine panel limited to IL‐6, IL‐8, and IL‐10. This targeted approach reflects the mechanistic emphasis of the study on labor‐associated immune activation, neutrophil recruitment, and counter‐regulatory signaling rather than an exhaustive assessment of the inflammatory network. Although other mediators such as TNF‐α or IL‐1β were not examined, the selected cytokines capture complementary aspects of pro‐ and anti‐inflammatory balance during the peripartum period. In addition, while covariate‐adjusted analyses were not the primary analytical strategy, sensitivity analyses adjusting for mode of delivery supported the robustness of the main inflammatory findings, particularly for IL‐6. Finally, although no adverse fetal or neonatal outcomes were observed in this cohort, the persistent postpartum inflammatory profile identified in obese women may have implications for maternal recovery and longer‐term health, underscoring the need for further investigation in larger and more comprehensive studies.

In summary, the present findings indicate that maternal obesity is associated with distinct inflammatory patterns during labor and the immediate postpartum period. Specifically, CRP increased postpartum only in obese women, while IL‐6 declined postpartum only in non‐obese women, suggesting differences in peripartum inflammatory resolution trajectories rather than a uniform pro‐inflammatory state. Despite the well‐established association between obesity and oxidative stress, no between‐group differences were detected in the redox markers assessed in maternal blood or placental tissue, indicating that within the scope of the evaluated markers, redox balance appeared to be maintained during pregnancy and delivery. By capturing time‐dependent changes in key inflammatory mediators using paired peripartum sampling, this study provides new insight into how maternal obesity modulates immune‐endocrine regulation around delivery. These findings may be relevant for understanding postpartum inflammatory dynamics in obese pregnancies and underscore the need for future studies to investigate the long‐term consequences of altered inflammatory resolution and to explore broader inflammatory and redox pathways in this growing population.

## Funding

This study was supported by Fundaçãode Amparo à Pesquisa do Estado de Minas Gerais (FAPEMIG) (APQ‐02018‐24), and Kinulpe Honorato Sampaio is a recipient of Conselho Nacional de Desenvolvimento Científico e Tecnológico (CNPq) (303206/2022‐5).

## Conflicts of Interest

The authors declare that there is no conflict of interest.

## Supporting information




**Supporting File 1**: aji70227‐sup‐0001‐tableS1.docx

## Data Availability

The data that support the findings of this study are available from the corresponding author upon reasonable request.
