# Supplementary material for: Maternal Obesity Modulates Postpartum Inflammatory and Hormonal Profiles, Without Detectable Differences in Tested Redox Markers
Source: Am J Reprod Immunol. 2026 Mar 17;95(3):e70227. doi: 10.1111/aji.70227 (PMC12993264; doi:10.1111/aji.70227)
Supplement: Supplementary file 1 — Supporting File 1: aji70227‐sup‐0001‐tableS1.docx [file AJI-95-e70227-s001.docx]

**Supplementary Table S1: Sensitivity analyses adjusted for mode of delivery (vaginal vs. cesarean section)**

| Parameter | Model | Effect of Time | Effect of Obesity | Time × Obesity |
| --- | --- | --- | --- | --- |
| Neutrophils | Unadjusted | 0.001 (ω² = 0.157) | 0.887 (ω² = 0.000) | 0.474 (ω² = 0.000) |
|  | Adjusted for mode of delivery | <0.001 (ω² = 0.173) | 0.981 (ω² = 0.000) | 0.377 (ω² = 0.000) |
| CRP (mg/L) | Unadjusted | 0.004 (ω² = 0.059) | 0.216 (ω² = 0.033) | 0.163 (ω² = 0.007) |
|  | Adjusted for mode of delivery | <0.001 (ω² = 0.083) | 0.269 (ω² = 0.017) | 0.205 (ω² = 0.004) |
| Interleukin-6 (pg/mL) | Unadjusted | 0.100 (ω² = 0.027) | 0.028 (ω² = 0.013) | 0.294 (ω² = 0.002) |
|  | Adjusted for mode of delivery | 0.012 (ω² = 0.078) | 0.236 (ω² = 0.030) | 0.145 (ω² = 0.016) |

Values represent p-values from two-way repeated-measures ANOVA models (time × obesity), with partial omega squared (ω²) indicating effect size. Sensitivity analyses were performed adjusting for mode of delivery (vaginal vs. cesarean section).
